# CRISPR Assays for Disease Diagnosis: Progress to and Barriers Remaining for Clinical Applications

**DOI:** 10.1002/advs.202301697

**Published:** 2023-05-10

**Authors:** Zhen Huang, Christopher J. Lyon, Jin Wang, Shuihua Lu, Tony Y. Hu

**Affiliations:** ^1^ National Clinical Research Center for Infectious Diseases Shenzhen Third People's Hospital Southern University of Science and Technology 29 Bulan Road Shenzhen Guangdong 518112 China; ^2^ Center for Cellular and Molecular Diagnostics Tulane University School of Medicine 1430 Tulane Ave New Orleans LA 70112 USA; ^3^ Department of Biochemistry and Molecular Biology Tulane University School of Medicine 1430 Tulane Ave New Orleans LA 70112 USA; ^4^ Tolo Biotechnology Company Limited 333 Guiping Road Shanghai 200233 China

**Keywords:** commercialization, CRISPR, diagnosis, nanotechnology, point‐of‐care

## Abstract

Numerous groups have employed the special properties of CRISPR/Cas systems to develop platforms that have broad potential applications for sensitive and specific detection of nucleic acid (NA) targets. However, few of these approaches have progressed to commercial or clinical applications. This review summarizes the properties of known CRISPR/Cas systems and their applications, challenges associated with the development of such assays, and opportunities to improve their performance or address unmet assay needs using nano‐/micro‐technology platforms. These include rapid and efficient sample preparation, integrated single‐tube, amplification‐free, quantifiable, multiplex, and non‐NA assays. Finally, this review discusses the current outlook for such assays, including remaining barriers for clinical or point‐of‐care applications and their commercial development.

## Graphical abstract

CRISPR assays that permit sensitive detection of various new targets continue to evolve and have exciting potential for future clinical and commercial applications. However, while much has been done to improve the performance and workflows of these assays, significant challenges remain for their widespread commercial and clinical adoption, although lessons from nucleic acid amplification assays could facilitate this process.

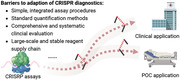

## 1 Introduction

Fast and accurate diagnosis is required for effective disease treatment, to evaluate treatment efficacy, and to prevent disease transmission.^[^ ^1^ ^]^ Nucleic acid amplification (NAA)‐based molecular diagnostics can directly detect pathogens^[^ ^2^ ^]^ and indicate drug resistance^[^ ^3^ ^]^ or specific pathophysiologic features (e.g., disease site).^[^ ^4^ ^]^ NAA diagnostics have gradually become the diagnostic reference standard for various acute and chronic conditionsparticularly infectious diseases,^[^ ^5^ ^]^ and their importance in disease diagnosis and prevention has been emphasized by their central role in the response to the COVID‐19 pandemic.^[^ ^6^ ^]^

However, this event has also highlighted that such diagnostics are poorly accessible in many global regions.^[^ ^7^ ^]^ Further, polymerase chain reaction (PCR), the most widely used NAA approach, can have unsatisfactory sensitivity with some specimen types, and its instrumentation and technical expertise requirements limit its utility in point‐of‐care (POC) applications. Sensitive real‐time quantitative PCR assays use NA‐specific dyes or fluorescent probes^[^ ^8^ ^]^ and the sensitivity of these assays can be further enhanced by the confinement effect of digital droplet PCR^[^ ^9^ ^]^ at the cost of increased complexity that limits their scope of utility. Recombinase polymerase amplification (RPA)^[^ ^10^ ^]^ and loop‐mediated isothermal amplification (LAMP)^[^ ^11^ ^]^ reactions developed as alternatives to PCR can rapidly and efficiently amplify NA targets at a constant temperature to dispense with the need for a thermocycler and expand their scope of use.^[^ ^12^ ^]^ This advantage, however, is counterbalanced by their potential requirements for high primer and enzyme concentrations and the use of denaturing agents and complex buffer systems, which may affect their sensitivity and specificity.^[^ ^13^ ^]^ New simple, rapid, and inexpensive methods that permit sensitive and specific NAA diagnosis are thus needed to address the increasingly urgent infectious disease threats.

New CRISPR (clustered regularly interspaced short palindromic repeats)‐based diagnostic (CRISPR‐Dx) assays can address several drawbacks of NAA‐based diagnostics that are of growing clinical importance.^[^ ^14^ ^]^ CRISPR systems are employed by bacteria and archaea as adaptive immune systems that employ guide RNA (gRNA) sequences encoded in the CRISPR locus to direct a CRISPR‐associated (Cas) endonuclease activity to recognize and cleave specific foreign NA sequences.^[^ ^15^ ^]^ Cas/gRNA complexes that employed synthetic gRNAs specific to target gene lesions were initially used for gene editing^[^ ^16^ ^]^ and then rapidly adopted to specifically amplify target signals in NA diagnostic assays to enhance their diagnostic sensitivity. Such CRISPR‐Dx assays are highly programmable and demonstrate high sensitivity and specificity to allow robust tracking of scarce NA targets in complex biological specimens, rendering them an extremely useful means to increase the sensitivity of suboptimal NAA diagnostic assays.

Extensive literature now describes CRISPR‐Dx applications for various disease, but there has been little discussion of the challenges that remain for their clinical translation following their initial analytical validation studies.^[^ ^17^ ^]^ These include generating streamlined universal sample preparation protocols, producing simplified and integrated assay procedures, establishing accurate biomarker quantification methods, developing integrated multiplex biomarker panels, and generating and refining new CRISPR‐Dx assays for non‐NA targets.

This review will provide an overview of the complete CRISPR‐Dx development process from the selection of the CRISPR system appropriate for a specific target type, through the steps required to establish a clinical application and describe barriers that may limit the development and commercialization of these applications and how to surmount them with novel approaches, particularly those based on micro‐ and nano‐technology.

## 2 CRISPR/Cas System Characteristics and their Application to CRISPR‐Dx Assays

### 2.1 CRISPR/Cas System Characteristics

**Figure 1:**
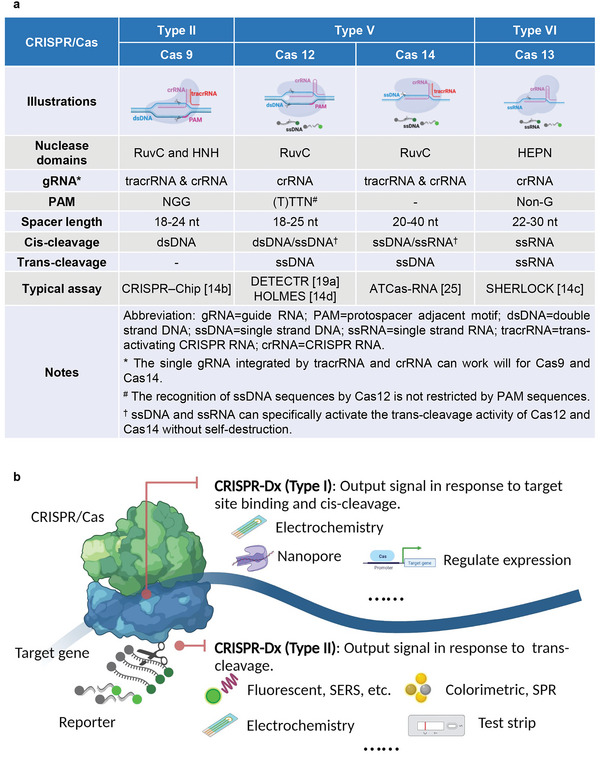
Summary of Class II CRISPR/Cas system characteristics and their use in proposed analytical applications. a) Characteristics of four Class II CRISPR/Cas systems frequently used in diagnostic applications. b) CRISPR assays are proposed for applications that employ CRISPR binding or cis‐cleavage activity (Type I) or CRISPR trans‐cleavage activity (Type II) for biomarker detection.

All current CRISPR‐Dx assays utilize Class II CRISPR/Cas systems that consist of type II (Cas9), type V (Cas12 and Cas14), and type VI (Cas13) CRISPR/Cas systems that employ single multidomain effectors.^[^ ^18^ ^]^ These CRISPR systems have distinct differences in their gRNA structures, protospacer‐adjacent motif (PAM) sequences, targeted NA types, and cleavage activities (**Figure** **1**a). Most CRISPR/Cas systems that have been reconstituted in vitro have similar features in common, with individual exceptions. These include the use of a gRNA‐mediated PAM search mechanism, a double‐strand helicase activity (not required for single‐strand NA targets), a stepwise gRNA‐DNA base pairing mechanism, and site‐specific *cis* cleavage activity, with or without a non‐specific *trans* cleavage function.^[^ ^19^ ^]^ For example, Cas12 and Cas13 employ a single gRNA^[^ ^19a,b^ ^]^ while Cas9 and Cas14 rely on complex gRNAs composed of *trans*‐activating CRISPR RNA (tracrRNA) and CRISPR RNA (crRNA) components,^[^ ^19c,19d^ ^]^ where the former serves as the handle of the Cas effector and the latter directs matching to the target sequence. However, a tracrRNA and crRNA pair can be integrated into a single gRNA to reduce the complexity of gRNA design for CRISPR/Cas9 and Cas14 systems. Structural and chemical gRNA modifications can also be used to significantly alter the nuclease activity or specificity of CRISPR/Cas effectors.^[^ ^20^ ^]^ For example, engineering a gRNA with a 3′ A‐rich 7‐nt DNA extension can increase the *trans* cleavage activity of LbCas12a 3.5‐fold over that obtained using a wild‐type crRNA.^[^ ^20f^ ^]^ Rational design of gRNAs can thus be an important means to improve detection sensitivity, particularly in the absence of a target NAA step.

Specific recognition of dsDNA targets by CRISPR/Cas9 and Cas12 systems is normally determined by recognition of a PAM motif (Figure 1a) that promotes dsDNA unwinding and hybridization of the remaining crRNA sequence with complimentary ssDNA sequence in the dsDNA target.^[^ ^19k^ ^]^ This PAM recognition feature reduces the risk of stable interaction with off‐target NA targets to maintain the high specificity of CRISPR/Cas system target recognition required for CRISPR‐Dx assays but also complicates gRNA design by limiting the number of potential target sites available for analysis. Screening can identify candidate sequences that contain PAM sequences, but when these are not available in a target region a PAM sequence can be introduced into an amplicon during the NAA process by incorporating it into one of its primers.^[^ ^21^ ^]^ In this approach, the gRNA recognizes a PAM sequence contributed by the primer but otherwise has minimal overlap with the primer sequence to prevent off‐target recognition of NAA artifacts. The design of gRNAs intended to detect specific single‐nucleotide polymorphisms (SNPs) is more challenging, as a PAM site must often overlap or be directly adjacent to the target SNP to achieve the sensitivity required to distinguish a single‐nucleotide mismatch. SNPs that create, alter, or destroy PAM sites can act as a sensitive means to detect these sequence variants as they can frequently exhibit strong differential recognition to identify strains with different phenotypes.^[^ ^21^, ^22^ ^]^ For example, our group has used this approach to detect SNPs in the SARS‐CoV‐2 spike regions of several variants of concern (S982A and D950N) that could distinguish specific variants (alpha, delta), after determining that all the analyzed variants of concern contained 2 to 5 stain‐specific mutations in potential CRISPR PAM motif or seed regions.^[^ ^21b^ ^]^ Screening efforts could also potentially identify new CRISPR/Cas effectors with different PAM sequence specificities to expand the number of available sequence variants that could be detected by this approach, as could approaches that modify Cas proteins to reduce PAM dependence.^[^ ^23^ ^]^

Sequence recognition specificities for all CRISPR/Cas Class II systems are gRNA‐dependent, but their recognized NA type and cleavage activity are Cas‐specific (Figure 1a) and can determine their potential applications in diagnostic approaches. Cas9 and Cas12 are typically used to recognize dsDNA targets, while Cas13 and Cas14 are primarily used to detect ssRNA and ssDNA targets, respectively, due to their inherent target preferences. Such preferences are not always absolute, however, as Cas12a has also been reported to possess ssDNA threading activity that is not PAM dependent,^[^ ^19^, ^24^ ^]^ and Cas14a1 activity can also be induced by RNA target sequences.^[^ ^25^ ^]^ NA conversion reactions, often used in a pre‐NAA step,  can also allow DNA‐specific CRISPR/Cas effectors to detect RNA targets.^[^ ^14c,d^ ^]^ For example, most CRISPR‐based COVID‐19 tests employ reverse transcription and NAA to convert SARS‐Cov‐2 RNA into dsDNA amplicons that are detected by Cas12a/gRNA complexes.^[^ ^26^ ^]^ Cas cleavage characteristics are not readily modifiable, and all Class II Cas proteins except Cas9 possess an ssDNA/ssRNA‐specific collateral nuclease activity that is induced following the recognition of a NA substrate containing a target sequence (Figure 1a). This secondary *trans*‐cleavage activity exhibits high efficiency but lack strick sequence specificity^[^ ^14^, ^19^, ^24^, ^27^ ^]^ and can cleave multiple copies of ssDNA during the time it takes a Cas/gRNA complex to cleave and release its bound target sequence. This activity can be employed as a signal amplification mechanism to increase the analytical sensitivity of CRISPR‐Dx assays, as discussed in more detail in the following sections.

Class II CRISPCas effectors employed in CRISPR‐Dx assays are continuously updated through screening or engineering approaches.^[^ ^18^, ^28^ ^]^ Since these effectors exhibit different structures and functional domains, molecular weights, editing modes and efficiencies, and target specificities^[^ ^29^ ^]^ a clear understanding of these properties is required when developing new CRISPR‐Dx applications.

### 2.2 CRISPR‐Dx Assay Subtypes

Current CRISPR‐Dx assays can be divided into two categories based on whether they do or do not rely on Cas *trans*‐cleavage activity (Figure 1b). Those that do not rely on Cas *trans*‐cleavage activity primarily rely on the programmable binding of the Cas/gRNA complex to recognize a specific target sequence^[^ ^14^, ^30^ ^]^ since this binding can be more rapid and specific than traditional NA probe hybridization and does not require subsequent wash steps that can increase variability. These methods often employ deactivated Cas9 (dCas9) protein, which can bind but not cleave its targeted DNA sequence, and detect the resulting Cas binding events through a variety of strategies. For example, some approaches monitor the change in the electrical characteristics of graphene‐based field‐effect transistors (Figure S1a, Supporting Information)^[^ ^14^, ^31^ ^]^ or nanopores (Figure S1b, Supporting Information)^[^ ^30a,b^ ^]^ caused by the selective hybridization of target DNA with dCas9. A sandwich‐type CRISPR/dCas9 lateral flow assay has also been employed to detect swine fever virus target genes (Figure S1c, Supporting Information).^[^ ^30c^ ^]^ CRISPR/Cas9 *cis*‐cleavage activity can also be used as a switch to initiate assay signal output through an alternate mechanism. For example, Cas/gRNA complex binding has been used to activate a toehold sensor to distinguish specific strains of the Zika virus.^[^ ^22^ ^]^ In this approach, the Cas/gRNA complex binds and cleaves a Zika target amplicon that contains a strain‐specific PAM site so that the resulting RNA transcripts generated from this template are unable to activate the toehold switch sensor to produce the colorimetric assay signal (Figure S1d, Supporting Information). Finally, while Cas9 and dCas9 proteins are often employed in these assay approaches, other class II CRISPR/Cas effectors can also be used in these assays as long as the assay readout is not affected by their *trans*‐cleavage activity.

CRISPR‐Dx assays that employ Cas proteins with *trans*‐cleavage activity are, however, more common than those relying only on the binding or *cis*‐cleavage activity of their target‐specific Cas/gRNA complex. This is primarily since the induction of their *trans*‐cleavage activity upon target binding can be used to efficiently degrade synthetic assay probes to produce a variety of signal readouts (fluorescence,^[^ ^14^, ^19^ ^]^ surface plasmon resonance,^[^ ^32^ ^]^ electrochemistry,^[^ ^33^ ^]^ etc.) to enhance assay sensitivity. The first reported CRISPR assay employed the RNA‐specific Cas13 for signal amplification to produce the SHERLOCK (specific high‐sensitivity enzymatic reporter unlocking) assay^[^ ^14c^ ^]^, which cleaved a quenched fluorescent oligonucleotide probe to produce an assay signal. This approach was subsequently modified for multiplex detection in SHERLOCK V2 by using three Cas13 variants with different trans‐cleavage sequence specificities and Cas12a, which recognizes DNA target sequences and was adapted to permit point‐of‐care detection using a lateral flow assay format.^[^ ^34^ ^]^ Another group independently developed DETECTR (DNA endonuclease‐targeted CRISPR trans reporter)^[^ ^19a^ ^]^ and HOLMES (one‐hour low‐cost multipurpose highly efficient system)^[^ ^14d^ ^]^ assays that employed Cas12a for signal amplification. These assays could rapidly and inexpensively detect DNA or RNA targets with attomolar sensitivity, and resolve single‐nucleotide sequence mismatches, and thus served as the template for the explosive growth of subsequent CRISPR assays that have employed biotechnology and micro/nanotechnology platforms to further enhance performance. However, several factors can still limit the utility of CRISPR diagnostics for specific applications.

## 3 Challenges of CRISPR‐Dx Assays

### 3.1 Extraction‐free CRISPR‐Dx Assays

**Figure 2:**
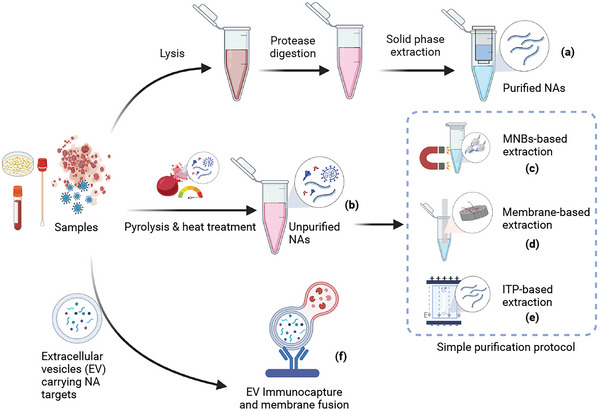
Nucleic acid (NA) release/isolation approaches proposed for CRISPR assays. a) Traditional NA isolation protocols are multi‐step and require technical expertise and expensive laboratory equipment. b) Extraction‐free NA extraction protocols often employ heat and chemicals to release NA from microbes or protein complexes for direct analysis without NA isolation or concentration. However, these procedures can be employed with c–e) rapid NA isolation protocols that do not require substantial operator effort or technical expertise. These include approaches that use c) magnetic nanobeads (MNBs), d) capture membranes, or e) isotachophoresis (ITP) to isolate NA from other sample components. f) Vesicle containment approaches can also be used to confine NA targets and CRISPR reagents in small volumes to enhance reaction kinetics and signal production, and thus target detection.

Rapid and efficient NA extraction remains a substantial bottleneck for on‐site NA detection approaches,^[^ ^35^ ^]^ including CRISPR‐Dx assays. Traditional NA extraction methods use multiple transfer steps that require additional equipment and technical expertise (**Figure** **2**a),^[^ ^35^, ^36^ ^]^ and automated NA extraction systems employed for rapid high throughput testing are expensive.^[^ ^36^, ^37^ ^]^ New approaches are thus needed to promote the adoption of CRISPR‐Dx assays, particularly for point‐of‐care applications.

CRISPR‐Dx assays have a high tolerance for biological matrices, permitting the use of protocols that use in situ reduction/heating reduction steps to release the NA content of a sample prior to its analysis in one‐tube reactions (Figure 2b). HUDSON (heating unextracted diagnostic samples to obliterate nucleases) uses chemical reduction and/or heat denaturation to lyse pathogens and inactivate sample nucleases within 30 min,^[^ ^38^ ^]^ and this protocol time can be shortened to < 10 min by modifying the lysis buffer conditions (Figure S2a, Supporting Information).^[^ ^39^ ^]^ This protocol is simple, employed in commercial kits,^[^ ^40^ ^]^ compatible with multiple clinical specimen types, and extensively validated in assays for various pathogens.^[^ ^38^, ^39^, ^41^ ^]^ However, its use in CRISPR‐Dx applications may reduce its ability to detect trace targets in complex matrixes since it lacks NA purification and concentration steps. Several methods have thus been developed to permit streamlined NA isolation from complex samples.

For example, nanoscale and microscale materials and technology applications can balance protocol constraints imposed when attempting to achieve high sensitivity with a simple and convenient process (Figure 2c–e). Nanoscale magnetic beads are frequently used in these approaches as they have large area‐to‐volume ratios, can be modified to have unique surface properties, and can be rapidly immobilized in targeted regions to support the rapid isolation and purification of NAs released into sample lysates (Figure 2c).^[^ ^42^ ^]^ The STOP (SHERLOCK testing in one pot) COVID‐19 assay, for example, uses nanoscale magnetic beads to eliminate the ethanol wash and RNA elution steps employing NA isolation from swab lysates to achieve a streamlined 15 min RNA extraction process (Figure S2b, Supporting Information).^[^ ^43^ ^]^ This procedure also concentrates magnetic beads that carry SARS‐CoV‐2 RNA from the entire swab into a single small‐volume STOP reaction to improve assay sensitivity.

NA isolation membranes generated using nanoscale or microscale fibers also have high surface area‐to‐volume ratios that can be employed for rapid and efficient NA isolation from lysed samples in high salt solutions through electrostatic interactions and hydrogen bonding (Figure 2d). NAs adsorbed onto these membranes remain stably bound during simple wash procedures used to remove contaminants and can be amplified in situ without elution to simplify NA concentration, purification, and amplification.^[^ ^44^ ^]^ For example, an RNA isolation procedure using an untreated nitrocellulose membrane can isolate NAs from inhibitors in a lysate using a 30 s process. Similarly, the self‐contained miSHERLOCK (minimally instrumented SHERLOCK) point‐of‐care COVID‐19 assay employs a 4 mm polyethersulfone membrane to isolate SARS‐COV‐2 RNA from 2 mL saliva through 3–6 min incubation step driven by gravity or capillary action (Figure S2c, Supporting Information).^[^ ^45^ ^]^

The methods listed above allow streamlined NA purification from large‐volume samples, but microfluidic methods permit rapid NA purification and concentration from small‐volume samples^[^ ^46^ ^]^ and can be integrated with simple NA isolation strategies to increase NA isolation efficiency (Figure 2e). For example, one study has reported that a microfluidic platform that uses isotachophoresis, a process in which the differential electrophoretic mobility of ionic species in strong applied electric field gradients can segregate different molecules,^[^ ^47^ ^]^ can isolate genomic DNA from nanoliter volumes of whole blood lysate within 3 min.^[^ ^47d^ ^]^ Further, integrating NA isolation procedures onto a microfluidic platform can permit the integration of the sample preparation, amplification, CRISPR reaction, and signal readout steps to streamline the assay and potentially improve its analytical and diagnostic performance (Figure S2d, Supporting Information).^[^ ^48^ ^]^

NA isolation and concentration procedures may also be unnecessary for some CRISPR applications. For example, extracellular vesicles (EVs), which are rich source of disease biomarkers, can be induced to fuse with liposomes loaded with CRISPR and other reagents to permit in situ isothermal detection of EV NA targets (Figure S2e, Supporting Information).^[^ ^49^ ^]^ The resulting fusion vesicles have nanoscale volumes to enhance assay kinetics and in situ detection of the assay's fluorescent signal. We used this approach to detect SARS‐CoV‐2 RNA in EVs directly captured from serum by EV‐specific antibodies conjugated to an assay well to permit rapid and streamlined COVID‐19 diagnosis, but this method could also be employed to detect NA biomarkers associated with other infectious, chronic, or malignant diseases (Figure S2e, Supporting Information).^[^ ^49a^ ^]^ This assay currently uses a procedure similar to ELISA but could be integrated into a microfluidic platform that uses other technologies (e.g., electric field‐driven microfluidics^[^ ^48a^ ^]^) to integrate steps and reduce the time required for the wash and membrane fusion steps of this procedure.

### 3.2 Assay Integration and Simplification

**Figure 3:**
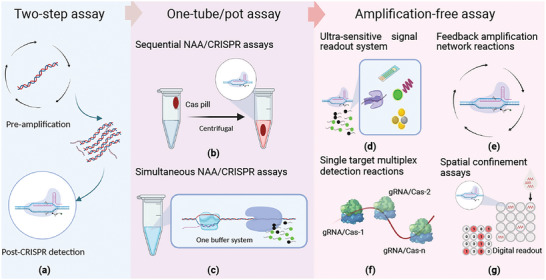
Simplification of CRISPR diagnostic workflows through system integration. a) Conventional CRISPR assays employ a two‐step reaction where target NAs are first amplified and then detected by target‐specific CRISPR activity to enhance sensitivity at the expense of increased assay completion time and cross‐contamination risk. b,c) Single‐tube assays can reduce these issues by b) sequential NAA/CRISPR reactions where assay reagents physically separated in the tube are sequentially introduced to the sample using controlled processes, or c) simultaneous NAA/CRISPR reactions that use same optimized reaction conditions to produce maximum assay signal. d–g) Non‐NAA CRISPR assays can streamline assay workflows, reduce costs, and greatly decrease the risk of contamination events, but require alternate readout systems, including d) ultra‐sensitive signal detection systems (electrochemical and nanopore sensor, etc.) and probes; e) feedback amplification systems (e.g., reactions where target recognition catalyzes gRNA production to establish a positive feedback circuit); f) multi‐Cas/gRNA systems, where a single target is recognized by distinct CRISPR/Cas complexes at different sites along its sequence; and g) spatial confinement assays, where sub‐nanoliter droplets confine reagents and single copy targets to enhance reaction kinetics, signal production, and target detection efficiency.

Most CRISPR‐Dx assays still use a standard two‐stage target detection paradigm (**Figure** **3**a),^[^ ^14^, ^19^ ^]^ first employing NAA to amplify the target sequence and then a separate CRISPR reaction to produce the assay signal. NAA increases the effective concentration of sequence in the CRISPR‐mediated detection reaction to permit the detection of scarce targets in the original sample. The primer matching required for NAA also increases target specificity, and this step can be coupled with other polymerases to alter which CRISPR/Cas effectors can be used for signal readout. However, this separate NAA step also increases assay performance time and the risk of cross‐contamination during transfer of amplified material to the CRISPR reaction. These downsides have driven efforts to develop CRISPR‐Dx assays that integrate the NAA and CRISPR reactions or dispense with the NAA step by using ultrasensitive signal detection methods.

#### 3.2.1 Single Tube CRISPR‐Dx Assays

Studies have examined methods that perform sequential or simultaneous NAA and CRISPR reactions in a single tube due to their ability to streamline assay performance and reduce the risk of contamination (Figure 3b,c). In sequential NAA/CRISPR assays, the NAA and CRISPR reagents are both present at sample addition but the CRISPR reagents are kept separate until introduced to the NAA reaction by a mechanism that does not require opening the assay tube (Figure 3b).^[^ ^26^, ^50^ ^]^ For example, one group immobilized Cas12a protein in the assay tube above the level of the NAA reaction and centrifuged these tubes after NAA reaction completion to initiate the CRISPR reaction (Figure S3a, Supporting Information).^[^ ^50a^ ^]^ Another group developed a multiphase aqueous reaction system where target NAA occurs in the high‐density phase at the bottom of the tube and amplicons dynamically diffuse to the low‐density upper phase to initiate the CRISPR reaction for fluorescent signal output (Figure S3b, Supporting Information).^[^ ^51^ ^]^ Both these approaches reduce cross‐contamination risk but have variable effects on reducing reaction delays caused by using separate NAA and CRISPR reactions.

Simultaneous NAA/CRISPR assays (Figure 3c) that integrate two reactions in a single buffer system also reduce cross‐contamination and are faster and less prone to disturbance than sequential single‐tube assay procedures. However, these assays can also be more challenging to establish due to differences in the buffer and temperature preferences of the NAA and CRISPR reactions and their opposing natures (template amplification versus cleavage). For instance, CRISPR/Cas systems exhibit robust activity in the temperature range employed for RPA‐mediated isothermal NAA reactions (37–42 °C), but the CRISPR reaction rate must often be carefully controlled to avoid excessive consumption of the RPA amplicon that can reduce its accumulation and overall assay sensitivity. One study addressed this by developing an all‐in‐one dual CRISPR/Cas12a (AIOD‐CRISPR) assay that employed gRNAs without PAM motifs (Figure S3c, Supporting Information).^[^ ^26b^ ^]^ These gRNAs allow their Cas12a/gRNA complexes to bind their NA targets and induce Cas12 *trans*‐cleavage activity to cut the quenched assay probe but not do not induce cis‐cleavage activity, preventing cleavage of the bound NA target and attenuation of the simultaneous NAA reaction.^[^ ^19^, ^52^ ^]^ These findings indicate the feasibility of using non‐canonical gRNAs to balance target amplification and cleavage by NAA and CRISPR reactions performed in parallel in a single tube. Subsequent studies have also used gRNAs that target suboptimal PAM sequences^[^ ^53^ ^]^ or that require photoactivation^[^ ^54^ ^]^ to prevent CRISPR reagents from prematurely degrading an NAA target sequence when both assays are performed in the same tube.

Simultaneous NAA/CRISPR assays that employ LAMP‐mediated NAA reactions are more difficult to develop since LAMP reactions operate at higher temperatures (55–65 °C) than tolerated by standard Cas proteins. Screening efforts have, however, identified CRISPR/Cas effectors that permit LAMP reactions to be used in simultaneous NAA/CRISPR assays.^[^ ^20^, ^43^, ^55^ ^]^ For example, STOP (SHERLOCK testing in one pot) employs LAMP but uses an AapCas12b effector that can maintain optimal nuclease activity over a wide temperature range (31–59 °C).^[^ ^43^ ^]^ Optimizing buffer composition can also be important when using simultaneous LAMP and CRISPR reactions. Reducing the reaction buffer Mg^2+^ ion concentration can permit LAMP reactions to operate at lower temperatures but also reduces CRISPR reaction efficiency.^[^ ^20f^ ^]^ The dWS‐CRISPR (digital warm‐start CRISPR) assay uses AsCas12a that has high trans‐cleavage activity at low Mg^2+^ ion concentrations to overcome this issue,^[^ ^55b^ ^]^ as well as phosphorothioate‐modified inner LAMP primers that reduce the LAMP reaction temperatures for improved CRISPR compatibility.^[^ ^55^, ^56^ ^]^

Such one‐pot assays thus can reduce contamination, streamline assay workflows, and/or shorten performance times, but can dramatically increase the complexity of method development. Optimized primers, gRNAs, and buffer conditions determined for these assays also frequently represent compromises that are not optimal for either reaction and which can reduce sensitivity.

#### 3.2.2 Non‐NAA CRISPR‐Dx Assays

CRISPR‐Dx assays that lack NAA steps can have more rapid and streamlined workflows and be less expensive than those that employ NAA, and are less subject to cross‐contamination risks, but require the use of strategies to improve their sensitivity. These include methods to increase their signal output by using highly sensitive readout systems or signal amplification reactions, by employing multiple CRISPR/gRNA complexes that recognize distinct regions of the target NA sequence, and by concentrating the NA target in a small assay volume to enhance the efficiency of the CRISPR reaction (Figure 3d–g).

**Ultra‐sensitive readout systems**. Standard CRIPR‐Dx assays detect fluorescent signals produced upon cleavage of a quenched target probe, which are produced in proportion to the target NA sequence detected in a reaction. These assays thus typically rely on an NAA step to produce sufficient concentrations of their NA target to achieve acceptable kinetics to detect trace amounts of NA targets. Sensitive detectors that capture weak CRISPR signals and advanced nano‐probes that produce high‐intensity signals have been employed to compensate for the weak signal produced by CRISPR reactions acting on scarce substrates (Figure 3d). Numerous studies have used electrochemical (Figure S1a, Supporting Information)^[^ ^14^, ^31^, ^33^, ^57^ ^]^ and nanopore (Figure S1b, Supporting Information)^[^ ^30^, ^58^ ^]^ sensor approaches to amplify weak electrical signals from CRISPR platforms to permit sensitive detection of scarce targets. These devices can be complicated and not readily portable, limiting their potential application in some settings, but microfabrication advances should permit the miniaturization and integration of these devices to address these concerns. CRISPR assay sensitivity can also be boosted through the use of high‐performance general‐purpose probes (e.g., surface‐enhanced Raman scattering probe^[^ ^59^ ^]^) that have been extensively validated on other sensing platforms or by using new probes that take advantage of the unique properties of metallic nanomaterials. For example, the fluorophore enhancing and quenching properties of gold nanoparticles^[^ ^60^ ^]^ can be exploited to design probes capable of sensitive detection of sub‐femtomolar level targets (Figure S4a, Supporting Information).^[^ ^32c^ ^]^ Nanoparticles have also been used to enhance assay sensitivity in standard and CRISPR‐based immunoassays since the substrate conversion rate of nanozymes comprised of catalytic metal nanoparticles can be higher than that of enzymes with the same activities.^[^ ^61^ ^]^ Gold/platinum nanozyme particles have been used to catalyze colorimetric substrate conversion to enhance the detection of samples that contain low concentrations of a target NA sequence.^[^ ^62^ ^]^ Further innovation in the design of CRISPR probes for ultrasensitive detection of scarce NA targets in complex samples may be required to facilitate the adoption of CRISPR assays that do not require NAA and can potentially be utilized in point‐of‐care settings and could benefit from approaches adopted for other diagnostic platforms.

**Feedback amplification systems**. Feedback networks can be used to exponentially amplify weak signals (Figure 3e), and at least one group has used a CRISPR‐mediated feedback loop to detect a low‐concentration NA target (Figure S4b, Supporting Information).^[^ ^63^ ^]^ This group designed a CRISPR/Cas‐only amplification network (CONAN) assay that employed two signal transducer Cas/gRNA complexes with different specificities (T1 and T2) and a dsDNA signal amplifier probe (A) consisting of a gRNA hybridized with two oligonucleotides with internal mismatches flanked by a quencher and fluorophore. In this assay, a Cas/gRNA specific for the target DNA sequence was pre‐assembled as the first transducer (T1) and its recognition of the assay target NA caused its trans‐cleavage activity to cleave the ssDNA regions of two oligonucleotides bound to the signal probe A to release a gRNA specific for this probe. Recognition of the target NA by T1 thus cleaved signal probe A allowing the formation of a second Cas/gRNA complex (T2) that recognized the signal probe A to generate fluorescent and further gRNA for T2 to produce a feedback loop to amplify the CRISPR activity and fluorescent signal induced by a low‐concentration target. In this approach, a single NA target could activate this positive feedback circuit that utilizes the excess signal probe A and Cas protein to produce an exponential increase in CRISPR activity and fluorescent signal and thereby achieve attomolar detection sensitivity without a NAA step.

**Multi‐Cas/gRNA assay systems**. CRISPR assays that employ multiple Cas/gRNA complexes specific for different sites on a NA target sequence represent a straightforward means to increase the signal accumulation rate to reach a detectable signal threshold in less time, as the CRISPR reaction rates in these assays are proportional to the number of Cas/gRNA complexes they use (Figure 3g).^[^ ^26^, ^64^ ^]^ CRISPR assays that employ multiple Cas/gRNA complexes can also reduce false‐negative arising from mutations in a target sequence, which can be essential when detecting RNA viruses with high mutation rates, such as SARS‐COV‐2. Further, similar to the basic approach used in feedback amplification systems, CRISPR/Cas effectors with different substrate specificities and cleavage activities can be used to establish cascade amplification systems to magnify the weak CRISPR activation induced by low concentration targets.^[^ ^34^, ^65^ ^]^ In one such cascade amplification strategy, Cas13a/gRNA recognition of a target microRNA sequence activates its *trans*‐cleavage activity to cleave a synthetic dsDNA hairpin oligonucleotide, allowing it to interact with a Cas14a/gRNA complex and activate its *trans*‐cleavage activity to cut a quenched fluorescent oligonucleotide probe. This cascade CRISPR/Cas (casCRISPR) system can achieve an ≈1000‐fold increase in target sensitivity versus a Cas13a assay that does not employ a Cas14a/gRNA‐mediated signal amplification mechanism (Figure S4c, Supporting Information).^[^ ^65^ ^]^ By comparison, SHERLOCK V2, which uses the CRISPR auxiliary protein Csm6 to enhance Cas13 signal, achieves an approximate 3.5‐fold signal increase over that oberserved with a standard Cas13 assay.^[^ ^34^ ^]^

**Spatial confinement assay systems**. Cas protein *trans*‐cleavage activity rates are proportional to the substrate concentration,^[^ ^14c,d^ ^]^ so approaches that increase the effective concentration in the vicinity of a Cas/gRNA complex can increase its cleavage activity to enhance assay sensitivity (Figure 3f). Digital CRISPR‐Dx (dCRISPR‐Dx) assays^[^ ^55^, ^66^ ^]^ inspired by digital PCR^[^ ^67^ ^]^ achieve this by employing an array of small‐volume CRISPR reactions to confine CRISPR reagents and their NA targets, thereby limiting their potential diffusion distance to increase their effective concentration.^[^ ^55^, ^66^ ^]^ NAA‐free dCRISPR‐Dx assays based on Cas12a and Cas13a (Figure S4d, Supporting Information),^[^ ^66b,e^ ^]^ respectively, have achieved a > 10000‐fold sensitivity increase using sub‐nanoliter volumes versus standard reactions performed in microliter volumes (Cas13a) or single‐molecule detection sensitivity (Cas12a). However, while this approach can achieve impressive detection sensitivities in the absence of NAA it also requires more technical expertise and instrumentation and has a lower throughput than other methods, which can limit its utility for clinical diagnostic applications.

Combinations of these or other strategies may further enhance the sensitivity of CRISPR‐Dx assays that lack a NAA step. For example, the FIND‐IT (Fast Integrated Nuclease Detection In Tandem) test uses eight gRNAs specific for a single target to activate Cas13a *trans*‐cleavage to truncate a primary reporter oligonucleotide that then serves to activate the nuclease of Csm6 to cleave and unmask the fluorescent activity of a quenched fluorescent secondary reporter oligonucleotide (Figure S4d, Supporting Information).^[^ ^68^ ^]^ This cascade‐based signal amplification strategy robustly detected ≈30 target RNA copies per microliter in a 20 min assay without a NAA step. Similarly, the SATORI (CRISPR‐based amplification‐free digital RNA detection) assay employs multiple Cas13a/gRNA complexes targeting sites of the SARS‐CoV‐2 N‐gene in a spatial confinement microchamber to achieve an ≈5 femtomolar limit of detection within a 5 min reaction time.^[^ ^66f^ ^]^ Rational integration of the above strategies can thus permit rapid and sensitive target detection without the cross‐contamination risk associated with assays that employ NAA steps. However, strategies employed to compensate for the loss of sensitivity due to the lack of a NAA assay step can introduce extra complexity and expense, and relative advantages and disadvantages associated with these strategies must be considered when choosing or designing a detection method.

### 3.3 Quantitative CRISPR‐Dx Assays

NA biomarker levels can indicate disease burden and its response to treatment,^[^ ^4^, ^69^ ^]^ but most current CRISPR diagnostics provide qualitative results. Standard curves generated using serial dilution standards can be used to provide relative quantification results^[^ ^26^, ^34^ ^]^ to track disease progression and predict outcomes,^[^ ^70^ ^]^ which could greatly facilitate the treatment regimen selection for disease management. However, standard curve results can have high variation when used to measure targets present at low abundance, and careful NAA optimization is required to avoid saturation when analyzing targets with large dynamic ranges.^[^ ^34^, ^70^ ^]^ Reliable quantification also requires the use of standards generated with target samples of known purity and concentration, which may not always be commercially available in a format suitable for such assays.

Standard curves are not required for quantitative dCRISPR‐Dx assays (Figure 3f)^[^ ^55^, ^66^ ^]^ that compartmentalize a reaction into tens of thousands of sub‐nanoliter‐sized reactions where the presence or absence of fluorescence in each droplet indicates the presence or absence of a target in that droplet (Figure S4c, Supporting Information). Absolute quantification is achieved by counting the number of positive reactions and applying Poisson statistics to determine the number of targets required to produce an oberved result. Excellent linear response has been reported between input and measured NA concentrations for the seven dCRISPR‐Dx assays cited in this review. These assays now require specialized equipment to generate and read assay signals from nanoliter droplets, rendering them less suitable for clinical laboratory applications, although portable microfluidic devices^[^ ^71^ ^]^ and smartphone‐based scanners^[^ ^72^ ^]^ could, in theory, be used to overcome these barriers to clinical translation.

### 3.4 Multiplex CRISPR‐Dx Assays

**Figure 4:**
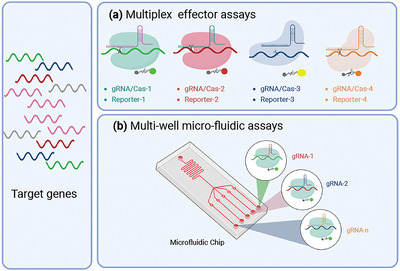
Strategies for multiplex CRISPR‐Dx assays. Two major multiplex CRISPR‐Dx assay approaches have been investigated to date. These are a) multi‐effector assays that employ Cas proteins with different trans‐cleavage sequence preferences to cleave distinctly labeled probes matching these specificities; and b) multi‐well assays where the assay chip wells contain to use the same CRISPR reagents bit different gRNAs, and specific targets are detected by the signal produced in distinct assay chip wells.

Multiplex CRISPR assays can enhance diagnostic performance or allow the simultaneous evaluation of related or divergent markers that can supply additional clinical information (co‐infection, multi‐drug resistance, etc.) at high throughput and reduced cost per target.^[^ ^73^ ^]^ Such multiplex reactions are possible in assays that employ a target‐specific CRISPR/Cas cis‐cleavage activity to cut a labeled amplicon.^[^ ^74^ ^]^ However, it is challenging to achieve this multiplex detection in assays that employ CRISPR/Cas *trans*‐cleavage activity for signal amplification since this activity often exhibits poor sequence specificity and can produce signal cross‐talk in multiplex assays by cleaving the probe assigned to a different Cas/gRNA complex.^[^ ^75^ ^]^ Nevertheless, at least two approaches have been attempted to develop multiplex diagnostic assays that use CRISPR/Cas *trans‐*cleavage activity for their signal readout (**Figure** **4**).

First, multiplex assays have been designed using Cas proteins with different trans‐cleavage substrate preferences (Figure 4a). SHERLOCK V2,^[^ ^34^ ^]^ the first multiplex CRISPR‐Dx assay, employed four CRISPR/Cas effectors (PsmCas13b, LwaCas13a, CcaCas13b, and AsCas12a) with differential *trans*‐cleavage specificity for reporter molecules containing different dinucleotide repeats (AU, UC, AC, and GA, respectively) and labeled with different fluorophores. This approach allowed SHERLOCK V2 to detect three ssRNA targets and one ssDNA target in one reaction (Figure S5a, Supporting Information). However, this strategy is constrained by the number of these Cas proteins and their relative specificity for their preferred substrates, which can produce signal overlaps to reduce assay specificity. Engineering Cas proteins to improve their preference for specific *trans*‐cleavage substrate sequences may be a potential way to solve this issue.

A simpler and less challenging multiplex approach avoids this specificity issue by splitting a diagnostic sample into different microwells containing CRISPR reagents with different target specificities, and results from each analyte are read by their array position (Figure 4b).^[^ ^76^ ^]^ This multiplex design can employ a different gRNA with the same Cas protein and reporter in each well to simplify assay design and optimization and its multiplexing capacity is limited primarily by the available volume of a diagnostic sample, particularly for very low concentration targets. CARMEN (combinatorial arrayed reactions for multiplexed evaluation of nucleic acids) demonstrates the current state‐of‐the‐art for such multiplexing platforms.^[^ ^77^ ^]^ In this assay platform, sample NAA reactions and CRISPR reagents are spiked with an array of distinct dyes that mark their specific contents, then separately emulsified to form one‐nanoliter droplets. These droplets are then added to a single tube and pipetted into a microwell array that can accommodate two droplets from the pooled emulsion tube at random to create all possible combinations. This chip is then sealed to isolate all the microwells, the identity of the two droplets in each well is determined by their color codes, and these droplets are then induced to merge by an applied electric field to simultaneously induce all these CRISPR reactions in a single‐chip multiplex digital droplet assay platform (Figure S5b, Supporting Information). This approach can robustly test > 4500 gRNA‐target pairs on a single array and has been employed to simultaneously discriminate 169 human‐associated viruses, comprehensively subtype influenza A strains, and identify mutations associated with HIV drug resistance.^[^ ^77a^ ^]^ Since CARMEN uses dye markers to determine the position of specific reactions on the array, additional gRNAs can be readily added or removed to accommodate new targets, as was done in a subsequent study designed to identify six SARS‐CoV‐2 variant lineages.^[^ ^77b^ ^]^ However, while CARMEN and other microarray methods have the potential to simultaneously detect hundreds of targets, further development is needed to simplify their equipment and analysis software to accommodate their routine use in clinical applications with large data volumes and complex outputs.

### 3.5 Non‐NA CRISPR‐Dx Assays

Sensitive detection and quantification of non‐NA biomarkers (such as proteins, small molecules, exosomes, ions, cells, etc.) can also aid in detecting early disease and accurately evaluating a disease response to treatment,^[^ ^78^ ^]^ as these biomarkers may be present at low concentration early in disease and following treatment initiation. Early detection and treatment may facilitate disease clearance or prevent disease‐associated pathology, which might not be possible at later disease stages. Sensitive treatment monitoring could also improve the evaluation of disease clearance to inform treatment decisions and permit early detection of drug resistance and disease recurrence. Assays that detect non‐NA targets, such as heavy metals, drugs, illegal additives, chemical pollutants, and other harmful substances,^[^ ^79^ ^]^ also have significant potential utility for environmental monitoring and food safety screening applications.

**Figure 5:**
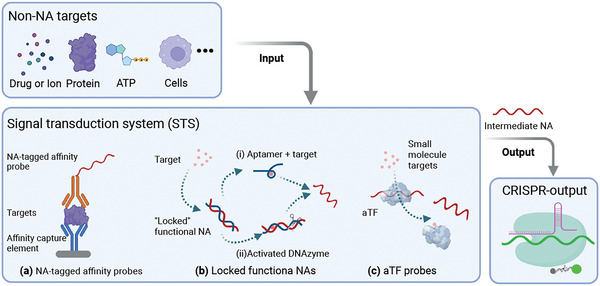
Non‐NA CRISPR diagnostic opportunities. Several CRISPR‐based strategies have been used to permit the ultrasensitive detection of non‐NA biomarkers and other molecules of interest. These include assays that use: a) NA‐tagged affinity probes (e.g., antibodies, receptors, ligands, and small molecules) that do not disrupt specific, high‐affinity interactions with their target molecules; b) functional NA/probe complexes (e.g., aptamers, DNAzymes, etc.) that release a NA target sequence upon their interaction with a biomarker target; and c) allosteric transcription factors (aTFs) that release a bound target NA sequence upon specific interaction with a small molecule ligand (e.g., metabolite, drug, etc.).

CRISPR/Cas system cannot directly detect non‐NA biomarkers, but several approaches have employed affinity probes tagged with CRISPR targets or reporters that release, unmask, or generate these sequences after specific interaction with their affinity targets to enable their detection (**Figure** **5**). Direct labeling strategies frequently use an affinity probe labeled with a NA sequence that can directly or indirectly serve as a CRISPR reaction target with or without an intervening NAA step (Figure 5a; Figure S6a, Supporting Information).^[^ ^80^ ^]^ Such approaches can yield a 10000‐fold sensitivity increase versus a conventional immunoassay.^[^ ^80d^ ^]^ NA‐tagged antibody probes are commonly employed for this purpose but other factors (e.g., receptors, ligands, or small molecules) that can be tagged with NAs without disrupting specific, high‐affinity interactions with their target biomarkers have similar prospects.

Affinity probes that employ functional NA sequences (aptamers or DNAzymes) have also been used to couple non‐NA target recognition with a CRISPR‐mediated signal readouts (Figure 5b). Aptamer‐based methods usually employ approaches where recognition of an affinity target either causes the aptamer to release a gRNA sequence or an amplification target for a subsequent CRISPR reaction.^[^ ^81^ ^]^ DNAzyme methods usually employ NAs that undergo a conformational change upon recognition of their affinity target to activate a masked DNAzyme activity that cleaves and releases a NA sequence that then serves as a gRNA or amplification target for a CRISPR reaction.^[^ ^81^, ^82^ ^]^ Notably, these methods can detect molecules not recognized by antibodies, as indicated by one of the first reports that used an aptamer/CRISPR assay to detect non‐NA targets (Figure S6b, Supporting Information).^[^ ^81c^ ^]^ In this study, aptamer binding released an aptamer‐bound ssDNA to induce Cas12a trans‐cleavage activity to cut and unmask a quenched fluorescent probe in the presence of ATP and Na^+^ ions. This approached achieved limits of detection of 0.21 µM in 15 min (ATP) and 0.1 mM in 35 min (Na^+^), with similar performance observed over a broad (25 to 37 °C) temperature range. This strategy has also been extended to detect bacteria,^[^ ^81^, ^82^ ^]^ exosomes,^[^ ^81b^ ^]^ proteins,^[^ ^81g^ ^]^ drugs,^[^ ^81i^ ^]^ and heavy metals.^[^ ^82^ ^]^ Functional NA sequences have significant inherent advantages when designing CRISPR assays that detect non‐NA targets, since functional NAs specific for distinct targets, including aptamers and DNAzymes, can be rapidly identified by in vitro selection approaches; readily modified to alter their stability, affinity, selectivity, and functional properties; and cost‐effectively synthesized at scale to facilitate assay development and deployment efforts.^[^ ^83^ ^]^ These properties can greatly reduce the time required to produce a new assay for non‐NA targets when compared to methods that employ antibodies or other affinity reagents, although usually at the expense of reduced sensitivity and specificity versus similar approaches that use antibodies for target recognition. CRISPR assays that employ functional NAs may also be negatively affected by components of biological specimens that may promote NA degradation or destabilize NA secondary structure to reduce NA stability,binding affinity, and selectivity.^[^ ^84^ ^]^

Allosteric transcription factors (aTFs) can also serve as affinity switches in non‐NA CRISPR assays as these DNA binding proteins undergo conformational changes after recognition of specific small molecules to alter their affinity for specific regulatory DNA sequences that can regulate gene transcription.^[^ ^85^ ^]^ This property can be used to release aTF‐bound NA sequences upon interaction with a small molecule ligands (drugs, metabolites, etc.) recognized by a specific aTF to induce CRIPSR signal in proportion to ligand concentration (Figure 5C; Figure S6c, Supporting Information).^[^ ^86^ ^]^ This design was used to establish CaT‐SMelor (CRISPR/Cas12a‐ and aTF‐mediated small molecule detector) assays for uric acid, tetracycline, and p‐hydroxybenzoic acid using different aTFs,^[^ ^86a^ ^]^ with follow‐up studies indicating that these assays had 10 and 2 nM limits of detection for uric acid and p‐hydroxybenzoic acid, and detected uric acid at levels found in human serum. In an adaptation of this design, SPRINT (SHERLOCK‐based profiling of in vitro transcription) assays employ aTF switches that recognize zinc and tetracycline to regulate in vitro transcription of RNA targets that activate a CRISPR/Cas13a‐based fluorescence detection system.^[^ ^86b^ ^]^ Similar to aptamers, aTFs can provide a sensitive and specific means to detect small molecules that are not detectable when using antibodies as affinity reagents. However, aptamers and aTFs have different strengths and weaknesses as affinity sensor molecules. For example, aTFs are more likely than aptamers to have higher affinity for a shared target molecule and to have greater stability in clinical specimens. Conversely, it is often possible to rapidly select aptamers with a desired specificity from a large synthetic library, while the selection of aTFs is limited to those that can be identified by screening microbial proteomes.

## 4 Summary and Outlook

CRISPR‐Dx assays have undergone a remarkable evolutionary expansion since their introduction seven years ago, although additional effort is still needed to accelerate their adoption as clinical applications. Much has been done to promote this adoption, including efforts to refine the workflow of these assays by streamlining sample preparation, simplifying assay procedures, establishing accurate methods for quantification and multiplex detection, and extending the scope of these assays to non‐NA targets. Microfluidic and nanomaterial applications have had a major role in these developments, and further developments and refinement of these applications could lead to more rapid commercialization of these assays and their adoption in clinical laboratories and point‐of‐care settings.

### 4.1 Future Direction: Development of CRISPR‐Dx Applications for Use in Low Resource Settings

Rapid and accurate disease diagnosis plays a critical role in improving patient outcomes, but almost half the global population has limited access to diagnostic assays.^[^ ^7^ ^]^ CRISPR‐Dx assays have potential to address the “last mile problem” of developing and supplying diagnostic assays that are usable in poor, rural, and marginalized global communities. However, few current CRISPR‐Dx meets the World Health Organization's ASSURED (Affordable, Sensitive, Specific, User‐friendly, Rapid and robust, Equipment‐free, and Deliverable to end‐users) criteria for POC tests^[^ ^87^ ^]^ that require such assays to be affordable, sensitive and specific, and deliverable to and usable in remote and resource‐limited areas.

Multiple studies have explored strategies to simplify CRISPR‐Dx assays,^[^ ^38^, ^43^ ^]^ but POC applications require systematic integration of CRISPR assay procedures into user‐friendly devices (e.g., microfluidic chips or lateral flow assays), not just the streamlining of individual assay steps. Microfluidic chips, lateral flow assay, or other integrated designs that require minimal training or user manipulation are needed to produce true sample‐to‐result diagnostics that can be used as POC tests. There have been attempts to develop such assays^[^ ^48^, ^88^ ^]^ and recent research also suggests that it may be possible to extend this development process to produce wearable devices that could diagnose or detect multiple distinct diseases or detect environmental exposures.^[^ ^88^, ^89^ ^]^ However, these efforts are still in the early stages.

Integrated diagnostic platforms intended for use in POC CRISPR‐Dx assays should also ideally have quantification and data‐sharing capabilities. Nanomaterial‐based colorimetric sensors can be easily integrated into CRISPR assays to allow qualitative visual analyses.^[^ ^26^, ^32^, ^34^, ^38^, ^90^ ^]^ This approach works well, reduces equipment requirements, and is easy to replicate in low‐resource settings but yields results that do not provide the precise and quantitative data that may be required to accurately evaluate disease burden and its response to treatment. However, this could be addressed by using inexpensive, widely available, and highly portable readout devices (e.g., smartphones)^[^ ^43^, ^45^, ^50^, ^64^, ^72^ ^]^ to quantify the colorimetric output of these assays. The network connections of these devices could also be used to directly share test results with health agencies and medical centers in real‐time to aid in epidemiological investigations, the formulation of disease prevention and control policies, and the practice of telemedicine in remote and resource‐limited areas. Several groups have also used widely available commercial handheld devices (e.g., fluorometers and glucose meters) to read signals produced upon CRISPR‐mediated cleavage of quenched fluorescent reporters^[^ ^81c^ ^]^ or by release of a conjugated enzyme that converts a reporter substrate into a material detected by the device (e.g., sucrose to glucose).^[^ ^91^ ^]^ Such approaches may permit existing technology to be leveraged to facilitate the adoption of POC CRISPR‐Dx assay applications that quantify NA and non‐NA biomarkers, although validation studies are necessary when applying these devices outside their intended use cases.

CRISPR‐Dx assays intended for use in low‐resource settings must also exhibit robust behavior when exposed to a range of transportation and storage conditions. Lyophilized CRISPR reagents stored at 4 °C for extended periods or at ambient temperature for several weeks exhibit only minor performance losses after rehydration.^[^ ^34^, ^45^, ^57^, ^88^ ^]^ However, further studies are needed to validate the performance of lyophilized CRISPR reagents after exposure to the more varied and extreme environmental conditions that may be encountered during transport or storage in resource‐limited settings that lack robust cold chains. Standard protocols for reagent stabilization need to be developed, validated, and adopted to facilitate the development of POC assays intended for use in these areas. Lyophilization and storage effects on assay performance should be considered in the early stage of assay development as should the shelf‐life of an assay in the absence of robust cold chain management in its intended use setting.

### 4.2 Shared Barriers to Clinical Translation: Key Milestones and Open Questions

Rigorous clinical validation studies required for clinical adoption have not been performed for most of the CRISPR‐Dx assays described to date. Many CRISPR‐Dx assay studies use simulated clinical samples to evaluate test performance and others often analyze samples from small retrospective case‐control cohorts, both of which can bias estimates of assay performance.^[^ ^92^ ^]^ However, a recent report has proposed essential characteristics required for clinical validation studies designed to evaluate the diagnostic performance of CRISPR‐Dx assays.^[^ ^90b^ ^]^ This includes the selection of an appropriate reference standard since a high‐sensitivity CRISPR assay may exceed the diagnostic performance of a single comparator assay and thus a composite reference standard that integrates results from several clinical tests or findings may be required to accurately identify all affected individuals in the analysis population. Standardized and validated CRISPR data analysis procedures are also urgently needed to allow uniform data reporting and comparison of results from different CRISPR assays.

Similar to NAA‐based diagnostic assays, CRISPR assay development would also benefit from streamlined patent licensing and market authorization procedures for CRISPR reagents and CRISPR‐Dx assays, and from the standardization and scaling of CRISPR reagent production. These changes would reduce assay development and production costs, reduce barriers to market entry, and diversify and stabilize supply chains, which can be a significant concern when attempting to meet growing demand, as observed in the recent pandemic. Streamlining all these processes also appears advisable, given the potential for CRISPR diagnostics to play an important role in rapid diagnosis of emerging diseases, as PCR‐based SARS‐CoV‐2 diagnostics did in the COVID‐19 pandemic after the FDA employed an Emergency Use Authorization policy to expand testing capacity.^[^ ^93^ ^]^

The modular and programmable nature of CRISPR system is an extremely valuable aspect of CRISPR assays, as this feature permits the specificity of an assay to be rapidly and easily modified by replacing its gRNA with one with a different sequence specificity. This should permit an assay optimized for use in one specimen type to be immediately adapted for another application upon demand, including the sensitive detection of new pathogen strains with different virulence or drug resistance profiles and new and emerging pathogens. This has significant implications for future disease outbreaks, epidemics, and pandemics since existing CRISPR reagent stockpiles be easily repurposed to address an emerging threat after the identification of a disease‐specific target sequence. In summary, CRISPR‐Dx applications are maturing to address the need for new assays that allow ultrasensitive detection of a variety of biomarker types. New applications have incorporated bioengineering, synthetic biology, nanomaterials, and microfluidics strategies to overcome specific challenges and diversify their application range. Challenges remain for the widespread commercial and clinical use of CRISPR‐based assays, but lessons learned from the history of NAA assays could facilitate the translation of these next‐generation molecular diagnostic tests, which could also find widespread applications in other areas, including food safety and environmental monitoring.

## Author Contributions

Z.H., C.J.L., and T.Y.H. conceived and designed this review. Z.H. and C.J.L. drafted the manuscript, and Z.H., C.J.L., J.W., S.H.L., and T.Y.H. provided critical revisions. All authors approved the final manuscript, and T.Y.H. was responsible for the decision to submit the manuscript.

## Conflict of Interest

J.W. is the founder of Tolo Biotechnology Company Limited, which holds the methodological patents of using Cas12 trans‐cleavage activity in nucleic acid detection. Other authors declare no conflict of interest.

## Supporting information

Supporting Information
