# Supplementary material for: CRISPR Assays for Disease Diagnosis: Progress to and Barriers Remaining for Clinical Applications
Source: Adv Sci (Weinh). 2023 May 10;10(20):2301697. doi: 10.1002/advs.202301697 (PMC10369298; doi:10.1002/advs.202301697)
Supplement: Supplementary file 1 — Supporting Information [file ADVS-10-2301697-s001.pdf]

ADVANCED SCIENCE
Open Access

Supporting Information

for *Adv. Sci.*, DOI 10.1002/advs.202301697

CRISPR Assays for Disease Diagnosis: Progress to and Barriers Remaining for Clinical Applications

*Zhen Huang, Christopher J. Lyon, Jin Wang, Shuihua Lu and Tony Y. Hu**

Supporting information

CRISPR assays for disease diagnosis: progress to and barriers remaining for clinical applications

Zhen Huang[1,2,3], Christopher J. Lyon[2,3], Jin wang[4], Shui hua Lu[1], and Tony Y. Hu[2,3†]

[1]National Clinical Research Center for Infectious Diseases, Shenzhen Third People's Hospital, Southern University of Science and Technology, Shenzhen, Guangdong, China
[2]Center for Cellular and Molecular Diagnostics, Tulane University School of Medicine, New Orleans, LA, USA
[3]Department of Biochemistry and Molecular Biology, Tulane University School of Medicine, New Orleans, LA, USA
[4]Tolo Biotechnology Company Limited, Shanghai, China.

[†]Correspondence to Tony Y. Hu: J. Bennett Johnston Building Rm 474, 1324 Tulane Ave, New Orleans, LA 70112. Tel: 504-988-5310. Email: tonyhu@tulane.edu.

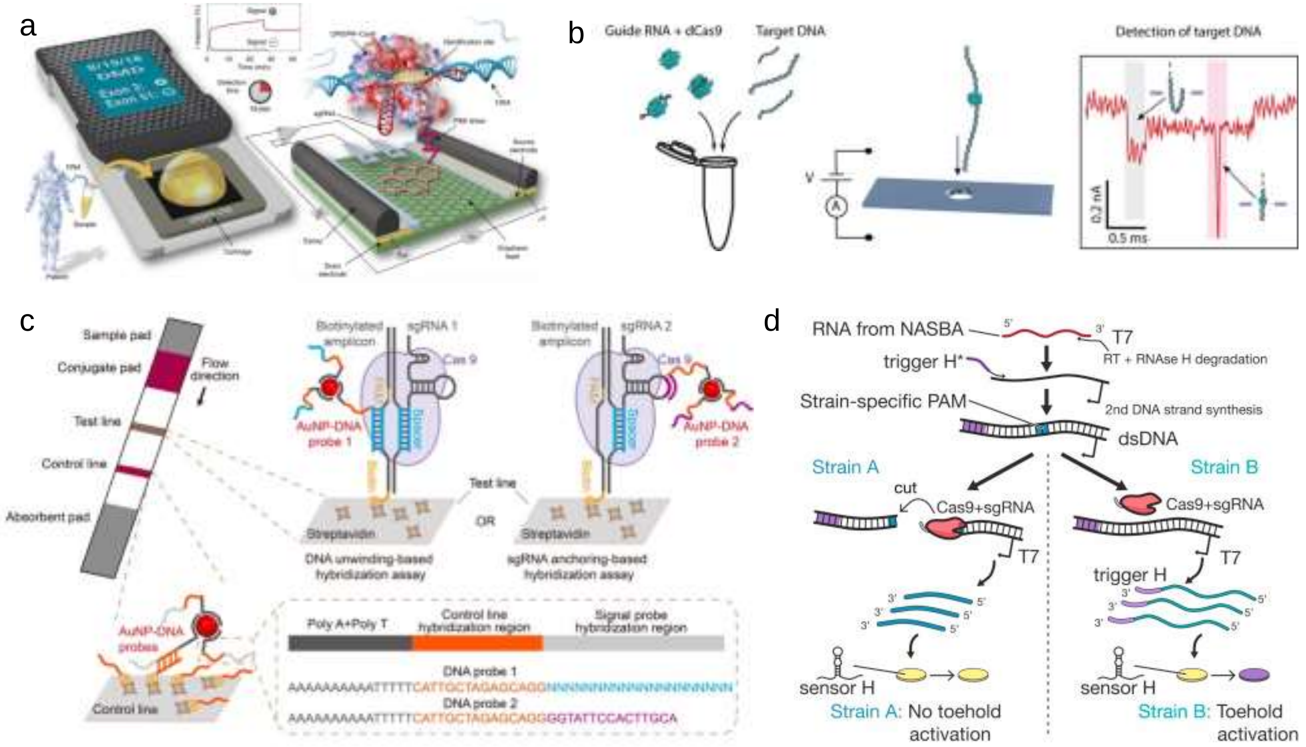

Fig. S1 CRISPR-Dx strategies that do not employ Cas trans-cleavage activity. (**a**) Selective binding of deactivated Cas9 (dCas9) to its target NA sequence alters the electrical characteristics of a graphene-based field-effect transistor to produce an electrical signal output. Adapted with permission. [**14b**] Copyright 2019, Springer Nature. (**b**) Binding of a dCas9/gRNA complex to its target NA sequence alters the electrical current produced when this NA interacts with a solid state nanopore to permit simple detection and quantification of these sequences. Adapted under the terms of the CC-BY-NC-ND license. [30b] Copyright 2018, American Chemical Society. (**c**) CRISPR/Cas9-mediated lateral flow nucleic acid assays employ dCas9/gRNA complexes to recognize biotinylated target amplicons bound to the streptavidin-conjugated test line, and detects this dCas9/gRNA binding using gold nanoparticle (AuNP) probes that bind either an unwound target sequence region or a gRNA hairpin region. Adapted with permission. [30c] Copyright 2020, American Chemical Society. (**d**) Cas9 cis-cleavage activity induced upon target NA binding truncates this NA to terminate the production of an RNA transcript that activates a toehold sensor that induces the production of a colorimetric assay signal. Adapted with permission. [22] Copyright 2016, American Chemical Society.

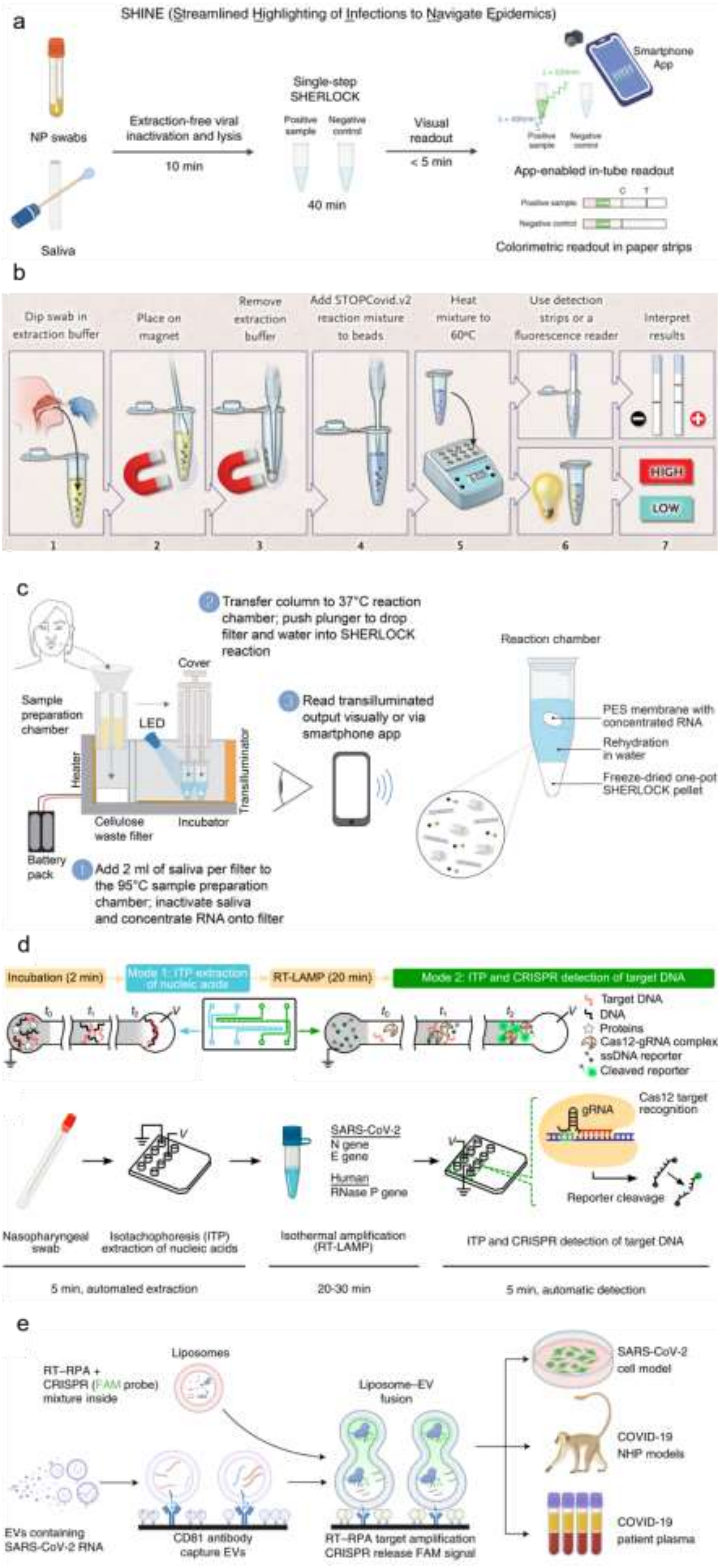
a
SHINE (Streamlined Highlighting of Infections to Navigate Epidemics)
NP swabs
Saliva
Extraction-free viral inactivation and lysis
10 min
Single-step SHERLOCK
Positive sample
Negative control
40 min
Visual readout
< 5 min
Smartphone App
App-enabled in-tube readout
Colorimetric readout in paper strips
b
Dip swab in extraction buffer
Place on magnet
Remove extraction buffer
Add STOPCovid.v2 reaction mixture to beads
Heat mixture to 60ºC
Use detection strips or a fluorescence reader
Interpret results
HIGH
LOW
1
2
3
4
5
6
7
c
Transfer column to 37°C reaction chamber; push plunger to drop filter and water into SHERLOCK reaction
Cover
Sample preparation chamber
LED
Heater
Transilluminator
Cellulose waste filter
Incubator
Battery pack
Add 2 ml of saliva per filter to the 95°C sample preparation chamber; inactivate saliva and concentrate RNA onto filter
Read transilluminated output visually or via smartphone app
Reaction chamber
PES membrane with concentrated RNA
Rehydration in water
Freeze-dried one-pot SHERLOCK pellet
d
Incubation (2 min)
Mode 1: ITP extraction of nucleic acids
RT-LAMP (20 min)
Mode 2: ITP and CRISPR detection of target DNA
Target DNA
DNA
Proteins
Cas12-gRNA complex
ssDNA reporter
Cleaved reporter
SARS-CoV-2
N gene
E gene
Human
RNase P gene
gRNA
Cas12 target recognition
Reporter cleavage
Nasopharyngeal swab
Isotachophoresis (ITP) extraction of nucleic acids
Isothermal amplification (RT-LAMP)
ITP and CRISPR detection of target DNA
5 min, automated extraction
20-30 min
5 min, automatic detection
e
Liposomes
RT–RPA + CRISPR (FAM probe) mixture inside
Liposome–EV fusion
EVs containing SARS-CoV-2 RNA
CD81 antibody capture EVs
RT–RPA target amplification CRISPR release FAM signal
SARS-CoV-2 cell model
COVID-19 NHP models
COVID-19 patient plasma

Fig. S2 Extraction-free CRISPR-Dx assays. (a) SHINE (Streamlined Highlighting of Infections to Navigate Epidemics), a sensitive and specific diagnostic tool that can detect SARS-CoV-2 RNA from unextracted samples, which improve HUDSON (heating unextracted diagnostic samples to obliterate nucleases) to rapidly inactivate viruses in nasopharyngeal swabs and saliva in 10 min. Adapted under the terms of the CC-BY license. [39a] Copyright 2020, Springer Nature. (**b**) The STOP (SHERLOCK testing in one pot) assay uses magnetic nanobeads to capture NA from lysed nasal swab specimens and directly analyzes these beads without an intervening NA elution and purification procedure. Adapted with permission. [43] Copyright 2020, Massachusetts Medical Society. (**c**) The miSHERLOCK (minimally instrumented SHERLOCK) assay uses polyethersulfone (PES) membranes to capture and concentrate NAs from heat-treated saliva samples after which these NA-enriched PES membranes are directly used for *in situ* NAA and CRISPR-mediated target detection. Adapted under the terms of the CC-BY-NC license.[45] Copyright 2018 The American Association for the Advancement of Science. (**d**) Microfluidic isotachophoresis devices can rapidly separate NAs from other components in nasopharyngeal swab samples prior to RT-LAMP NAA and CRISPR detection. Adapted under the terms of the CC-BY-NC license. [48a] Copyright 2020 National Academy of Sciences. (**e**) Liposomes loaded with CRISPR reagents can be induced to fuse with extracellular vesicles (EVs) directly captured from plasma samples by EV-specific antibodies to permit *in situ* detection of CRISPR signal produced by target-positive EVs. Adapted with permission. [49a] Copyright 2021, Springer Nature.

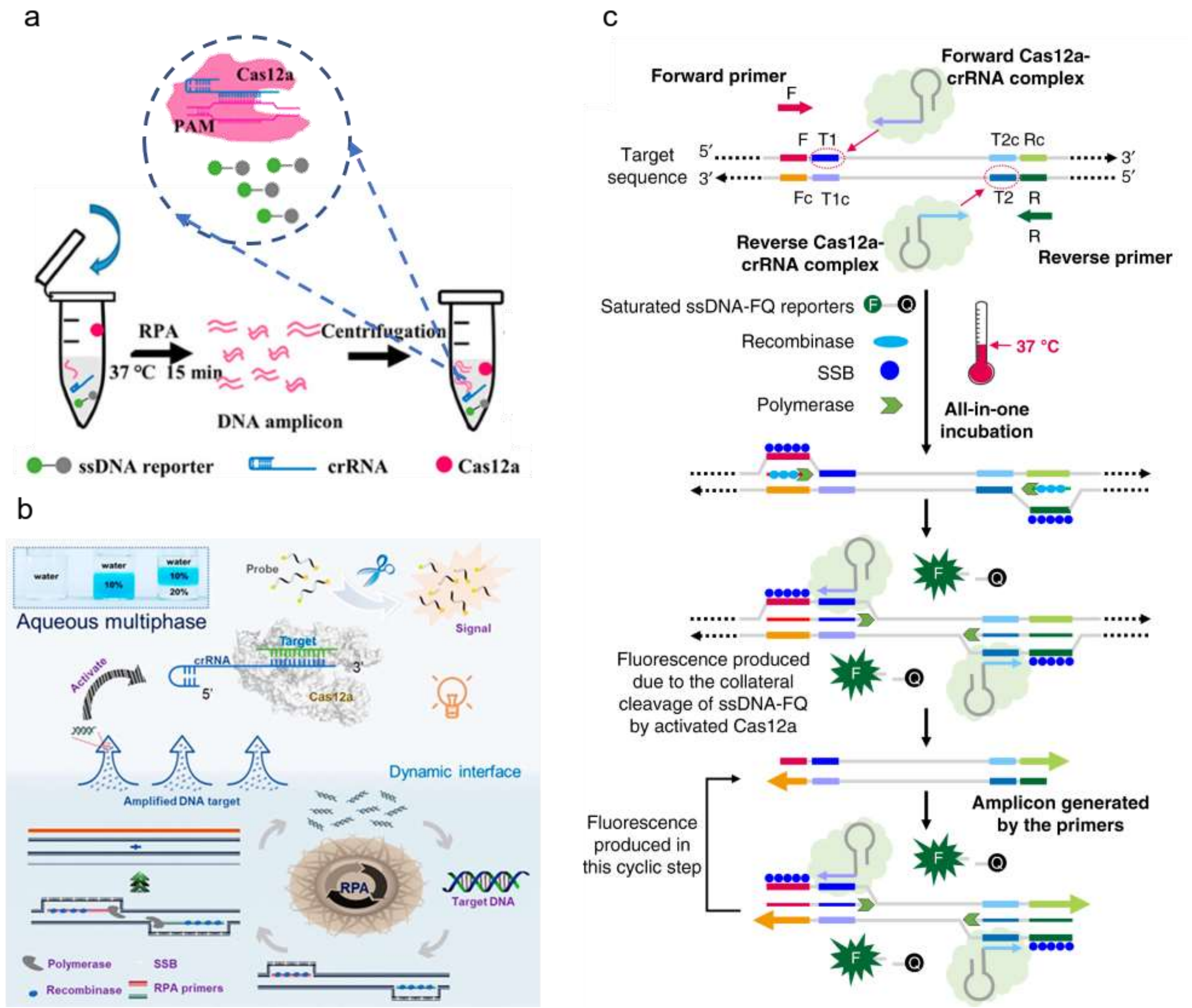

Fig. S3 Single closed-container CRISPR assay strategies. (**a**) Cas12aVDet (Cas12a-based Visual Detection) assays use centrifugal force to introduce CRISPR reagents pellets adhered side of the assay tube completed NAA reactions performed in the same tube. Adapted with permission. [50a] Copyright 2016, American Chemical Society. (**b**) Dynamic multiphase NAA/CRISPR reaction systems employ a density gradient to separate these reactions, which subsequently mix as amplicons produce in the high density NAA phase (bottom) diffuse to the low density CRISPR phase (top). Adapted with permission. [51] Copyright 2016, American Chemical Society. (**c**) An all-in-one dual CRISPR-Cas12a (AIOD-CRISPR) assay performed the NAA and CRISPR reactions in an adjusted reaction buffer, using gRNAs without PAM sites to prevent the Cas12a cis-cleavage activity from consuming the target NA amplicons and stalling the NAA reaction. Adapted under the terms of the CC-BY license. [26b] Copyright 2020, Springer Nature.

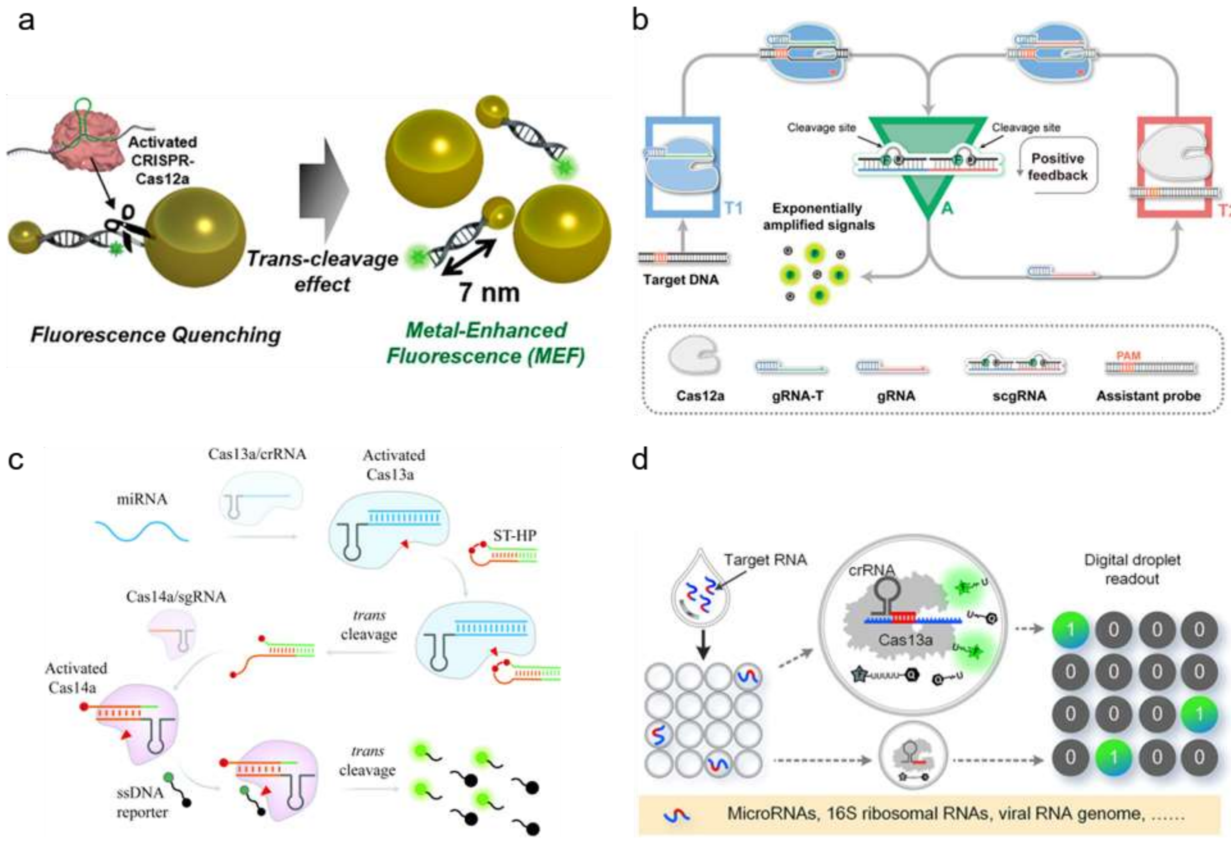

Fig. S4 Strategies used to generate sensitive Non-NAA-coupled CRISPR assays. (**a**) A probe design that employed metal nanoparticles to quench the activity of intact reporter and enhance the fluorescence signal of the cleaved reporter has reported to increase CRISPR assay signal-to-noise ratio to enhance assay sensitivity with NAA. Adapted with permission. [32c] Copyright 2021, American Chemical Society. (**b**) CONAN (CRISPR-Cas–only amplification network) assays use target recognition to cleave ssDNA regions on a quenched fluorescent probe to produce signal and release a gRNA targeting this probe to produce an autocatalytic positive feedback circuit resulting in exponential signal amplification without NAA. Adapted under the terms of the CC-BY-NC license.[63] Copyright 2021, The American Association for the Advancement of Science. (c) The cascade Cas13a–Cas14a (casCRISPR) system for miRNA detection. Cas13a/gRNA recognition of a target microRNA sequence activates its trans-cleavage activity to cleave a synthetic dsDNA hairpin oligonucleotide, allowing it to interact with a Cas14a/gRNA complex to activate its trans-cleavage activity to cut a quenched fluorescent oligonucleotide probe. Adapted with permission. [65] Copyright 2021, Royal Society of Chemistry. (**d**) Digital CRISPR-Dx assays separate the reaction volume into tens of thousands of nanoliter-sized reactions containing single or no copies of the NA target to increase the relative efficiency of CRISPR reactions in the constrained volume of the target-positive droplets. Adapted with permission. [66b] Copyright 2021, American Chemical Society.

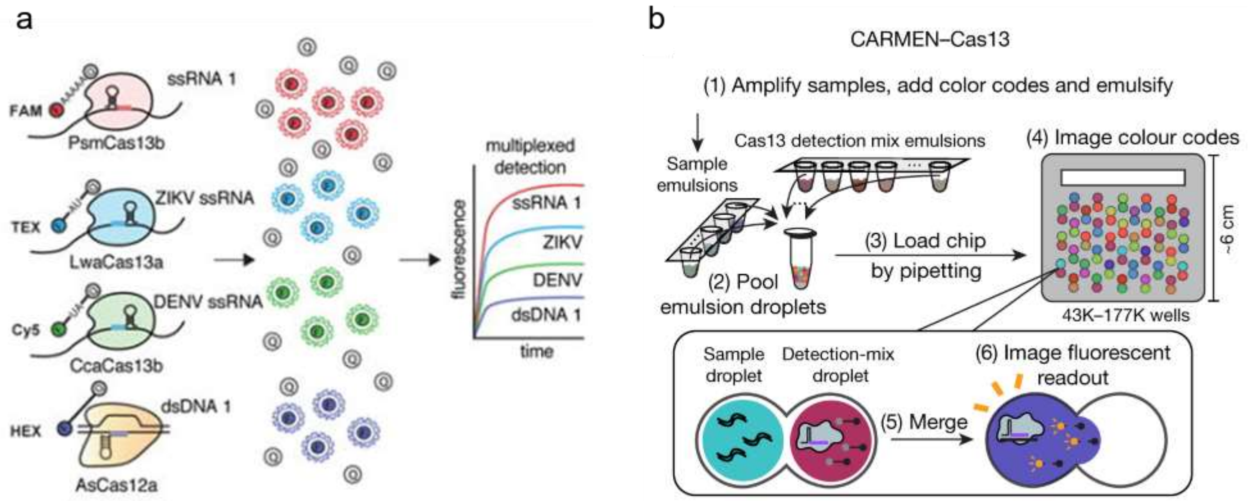

Fig. S5 Multiplex CRISPR assay methods. (**a**) SHERLOCK (specific high sensitivity enzymatic reporter unlocking) V2 employs four CRISPR/Cas effectors with different trans-cleavage substrate preferences to truncate matching reporter oligonucleotides labeled with four distinct quenched fluorophores. Adapted with permission. [34] Copyright 2018, The American Association for the Advancement of Science. (**b**) CARMEN (combinatorial arrayed reactions for multiplexed evaluation of nucleic acids-Cas 13) assays spike different NAA reactions and CRISPR reagents distinct dyes that mark their specific contents, then emulsify these samples and transfer them into a single tube before transferring these 1 nanoliter droplets into array wells that can contain accommodate only two droplets to randomly produce all possible sample-reagent combinations. Chips are then sealed and the droplet present in each well are identified by their color codes, and then induced to fuse by an applied electric field to simultaneously initiate CRISPR reactions in all wells. Adapted under the terms of the CC-BY license. [77a] Copyright 2020, Springer Nature.

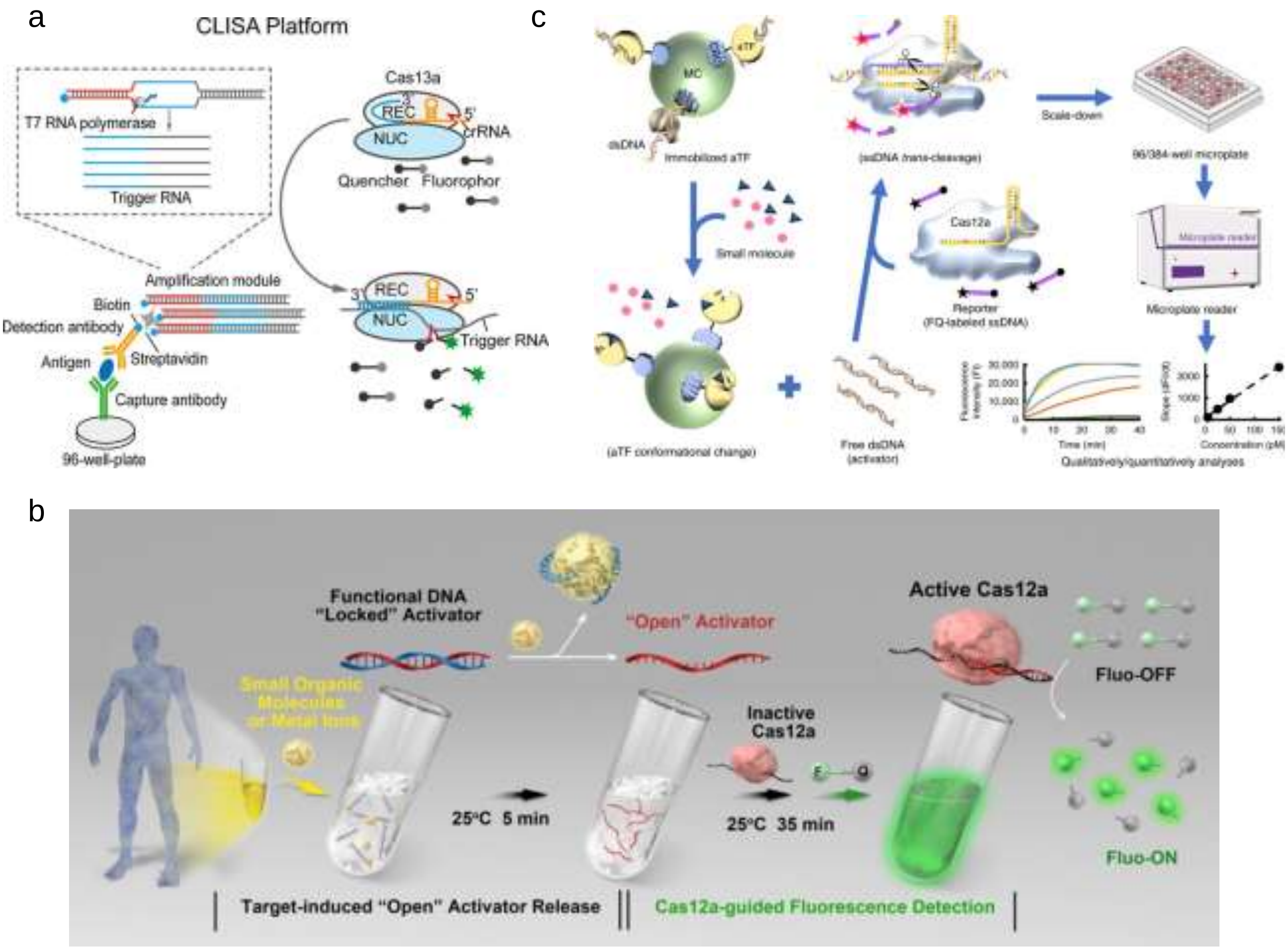

Fig. S6. CRISPR strategies for non-NA biomarker assays. (**a**) CRISPR/Cas13a signal amplification linked immunosorbent assay (CLISA) strategies use NA-tagged detection antibodies confer a NA signal that can be detected using standard NAA/CRISPR assay methods. Adapted with permission. [80a] Copyright 2019, American Chemical Society. (**b**) Functional DNAs that specifically bind small molecules and undergo conformational changes can be employed as sensor switches by releasing a NA sequence that can activate a CRISPR reaction. Adapted with permission. [81c] Copyright 2020, American Chemical Society. (**c**) Allosteric transcription factors (aTFs) specific for small molecules can also serve as sensor switches, since aTF/NA complexes introduced into an assay reaction release their bound NA in the presence of these molecules to serve as targets for a CRISPR assay readout. Adapted under the terms of the CC-BY license. [86a] Copyright 2019, Springer Nature.
